# Physical Culture in Education

**Published:** 1918-01-12

**Authors:** 


### PHYSICAL CULTURE IN EDUCATION.*

### I. Greek, French, and German Systems.

It is certain that from the most remote antiquity youths and men have delighted in the cultivation of physical prowess. Swiftness of foot and strength of arm were in primitive communities an essential to existence, and it is not until we come upon a more advanced condition of social organisation that we find them regarded as something more than utilitarian accomplishments. The famous Olympic games of the Athenians were at first of a Spartan type, testing the endurance with a view to war, and though they never entirely lost this character—for in the ancient world wars were as frequent as they were sudden—later innovations modified their purely military character. Thus, besides the Pancration, a combination of rough-and-tumble boxing and wrestling, were introduced foot-races, and the Pentathlon, in which were combined leaping, quoit-throwing, javelin-throwing, running, and wrestling. The youths admitted to these contests underwent for ten months a severe training in the gymnasia at Elis, and had to qualify in the preliminary heats before admission to the actual contests. Nor was the athletic training of those who did not wish to enter for the Olympian games neglected. The Athenian boy went to school daily, taking his meals at home, and, accompanied by his attendant slave, the pedagogue, was taken after his lesson in literature and music to the palestra for his athletics. Here he spent from three to five hours in open-air exercise. Sprinting and running, casting the javelin and discus, boxing and punching the *Korykos* or bag, were all studied and practised in turn, and the bath and massage concluded the day. The Olympian Pentathlon, or all-round contest, prevented over-specialisation, embodying, as Jebb finely says, the "central expression of the Greek idea that the body of man has a glory as well as his intellect and spirit, that body and mind should alike be disciplined, and that it is by the harmonious discipline of both that men best honour Zeus."

Let us now turn to modern times and note how this Greek ideal has been recognised—slowly, it is true—and is now to an increasing extent permeating the life of Western peoples. England, because of her love of field sports, has never been a pioneer in pure gymnastics, whilst the Teuton mind, tolerant of command and enamoured of mechanical exactitude, led the way in those forms of mass exercise which depend for their most impressive effect upon the precision and uniformity of the simultaneous actions of a large body of men. It is to these methods that modern physical education must look for one of the most powerful influences in its development. In the year 1774 Basedow founded at Dessau the Philanthropinum, to realise Rousseau's method of Nature "so that the training of the mind and the body shall serve to assist one another." In educating the sons of the burghers he employed the knightly exercises of riding, fencing, vaulting, and dancing; he also drew some exercises from popular sports, such as rowing, swimming, and games of ball, and it was from his influence and inspiration that later teachers received their inspiration. Another pioneer was Friedrich Ludwig Jahn, who was born in 1778; and his restless, intolerant disposition carried him from University to University, at each of which he devoted himself to the training of youths in manly sports and to the publication of works destined to arouse the then latent national spirit. His gospel, that of making physical training a dominant force in national regeneration, was a new one, and, like all innovations, was viewed askance by the official mind. In the spring of 1811 he opened his first training ground in a pine forest. From the first, vigorous and warlike games were adopted, and occupied a prominent place in the exercises. In a year the number of athletes rose to 500.

The formation of gymnastic societies continued to grow. In 1861, at the greater tournaments, the exercises, which continued for six days, opened with a mass drill of 20,000 men. Although the societies, drawn from distant lands, had never performed together before, the exhibitions were faultless. Great emphasis was placed upon mass work, singing accompanied many of the exercises, and everything was done to secure promptness and an aggressive carriage of the body.

Gymnastic instruction was made compulsory in many of the elementary schools in 1862; specially trained teachers (of whom before the war there were 1,500 in Berlin alone) adapting the exercises to the age and sex of the pupils. The youngest, ranging from six to ten years of age, were trained in a great variety of simple movements, games, marching, jumping, and climbing exercises. These were more complex and difficult for advancing age, and the expertness of the boys in the upper classes was often astonishing. Outdoor games have a certain place in the system, but it must be remembered that military service in Germany absorbs the energies of the best years of the young man's life.

### The War of the Systems in France.

In France the devastation of the Napoleonic wars, in which over 1,000,000 young men had met with an untimely death, left the nation in such an exhausted state that all attempts to introduce military gymnastics were unsuccessful. As late as 1867 one-third of the recruits had to be rejected on the ground of unfitness, and it was not till after the war of 1870 that France awoke to the vital importance of a system of physical training. Many parties arose, each advocating a different system. Some held that the introduction of field sports on the English model was what was needed, others advocated Swedish gymnastics, whilst a third party sought to discover the laws of movement, and to work from them a national system that would emphasise self-expression by harmonious movements, grace, and utility.

To each school was accorded a certain measure of support. Swedish gymnastics were at first introduced in their entirety at the Army Training College of Joinville, but were later modified by the characteristically French *savate*, rifle drills, boxing, and jiu-jitsu. About the same time English sports were introduced, and had at the outbreak of war been brought to a high state of perfection. Tennis especially has always been a game popular in France. As early as 1637 the master of Charterhouse School, after a visit to Paris, wrote: "The country is sown with tennis grounds. They are more numerous even than churches." Certainly the most interesting development in France has been that of eurythmics, with which the name of Dalcroze is associated. But other workers before Dalcroze had emphasised the fact that to secure adequate balance between opposing sets of muscles movements must be dynamic, and not static, jerky, or continuous, and the extremities of the limbs must describe in space sinuous lines, and not sharp angles, approaching in direction an ellipse, a spiral, or a figure of eight.

* *Exercise in Education and Medicine.* By R. Tait McKenzie, M.D. W. B. Saunders Company.

